# Application and prospect of ROS-related nanomaterials for orthopaedic related diseases treatment

**DOI:** 10.3389/fchem.2022.1035144

**Published:** 2022-10-05

**Authors:** Wenbo Yang, Qianwen Zeng, Qing Pan, Wei Huang, Hongzhi Hu, Zengwu Shao

**Affiliations:** ^1^ Department of Orthopaedic Surgery, Union Hospital, Tongji Medical College, Huazhong University of Science and Technology, Wuhan, China; ^2^ School of Nursing, Tongji Medical College, Huazhong University of Science and Technology, Wuhan, China

**Keywords:** nanomaterials, ROS regulation, therapy, review, orthopaedic related diseases

## Abstract

The importance of reactive oxygen species (ROS) in the occurrence and development of orthopaedic related diseases is becoming increasingly prominent. ROS regulation has become a new method to treat orthopaedic related diseases. In recent years, the application of nanomaterials has become a new hope for precision and efficient treatment. However, there is a lack of reviews on ROS-regulated nanomaterials for orthopaedic related diseases. Based on the key significance of nanomaterials for the treatment of orthopaedic related diseases, we searched the latest related studies and reviewed the nanomaterials that regulate ROS in the treatment of orthopaedic related diseases. According to the function of nanomaterials, we describe the scavenging of ROS related nanomaterials and the generation of ROS related nanomaterials. In this review, we closely integrated nanomaterials with the treatment of orthopaedic related diseases such as arthritis, osteoporosis, wound infection and osteosarcoma, *etc.*, and highlighted the advantages and disadvantages of existing nanomaterials. We also looked forward to the design of ROS-regulated nanomaterials for the treatment of orthopaedic related diseases in the future.

## 1 Introduction

The common orthopaedic diseases mainly traumatic injury orthopaedic disease, metabolic immune orthopaedic disease and abnormal proliferative orthopaedic disease. Some other diseases including prosthesis/wound infection and wound repair are also closely related to orthopaedics. Traumatic orthopaedic disease includes fracture, osteonecrosis, bone defect and so on (Einhorn and Gerstenfeld, 2015; El-Rashidy et al., 2017; Lespasio et al., 2019). Metabolic immune orthopaedic diseases include osteoporosis, osteoarthritis, rheumatoid arthritis, *etc.* (Scott et al., 2010; Pereira et al., 2015; Zhao et al., 2018a). Abnormal proliferative orthopaedic diseases are represented by hyperostosis and some kinds of bone tumors. However, an orthopaedic disease may correspond to different classifications, such as a bone tumor that may cause bone defects after resection (Zhao et al., 2018b). In general, clinicians will choose surgical treatment for local bone lesions and medical treatment for systemic bone lesions. However, it should be pointed out that for the current various orthopaedic diseases, almost none of the diseases have a treatment plan that can make the patient recover back to “total normal state”. Most patients show dysfunction or morphological changes after treatment, and may even have no obvious therapeutic effect. This has prompted us to explore mechanisms and innovate treatment options for a variety of orthopaedic diseases.

The occurrence of orthopaedic diseases is usually due to the disturbance of local metabolic level, in which REDOX reaction plays a great role. Many metabolic processes are inseparable from REDOX reactions. Common REDOX reactions related to metabolism include citric acid cycle, electron transfer and so on (Akram, 2014; Garrido-Pérez et al., 2020). In the study of the regulation of metabolic process, there is a kind of REDOX reaction intermediates-free radicals has attracted more attention. Free radicals are associated with a variety of biological processes, including aging, *etc.* (Harman, 2003). Reactive oxygen species (ROS), lipid free radicals are common free radicals (Valko et al., 2006; Matsuoka and Yamada, 2021). Their essence is that the molecules contain unpaired electrons and have high reactivity. ROS, as a major free radical type, plays an important role in regulating various physiological and pathological processes in cells and organisms, and has become one of the most cutting-edge and important research fields in many orthopaedic diseases. Generally speaking, ROS can be divided into endogenous and exogenous sources. Under normal circumstances, ROS produced spontaneously by cells are endogenous ROS, such as those produced in the process of oxidative phosphorylation in mitochondria (Berry et al., 2018). ROS increased by treatment, environmental exposure and other factors are generally exogenous ROS, including ROS caused by exposure to some nanoparticles (NPs), exposure to ionizing or non-ionizing radiation, chemotherapy drugs and microbial infection (Rimal et al., 2005; Wang et al., 2019; Li et al., 2020a; Ludovici et al., 2020; Golovynska et al., 2022). Under normal conditions, ROS plays an important role in the regulation of metabolism, but excessive ROS usually leads to cell damage and even apoptosis (Madreiter-Sokolowski et al., 2020; Tsubata, 2020). ROS is associated with the development and prognosis of many orthopaedic diseases, such as osteoporosis, osteoarthritis and bone defects (Ilyas et al., 2019; Li et al., 2020b; Zhou et al., 2022a). ROS has also been used as a therapeutic target or treatment for many orthopaedic diseases, such as osteosarcoma treated by photodynamic regimens and osteoarthritis treated by reducing ROS levels (Ding et al., 2021; Lu et al., 2021). The controlled regulation of ROS may be the key to breakthrough in the treatment of orthopaedic diseases.

**FIGURE 1:**
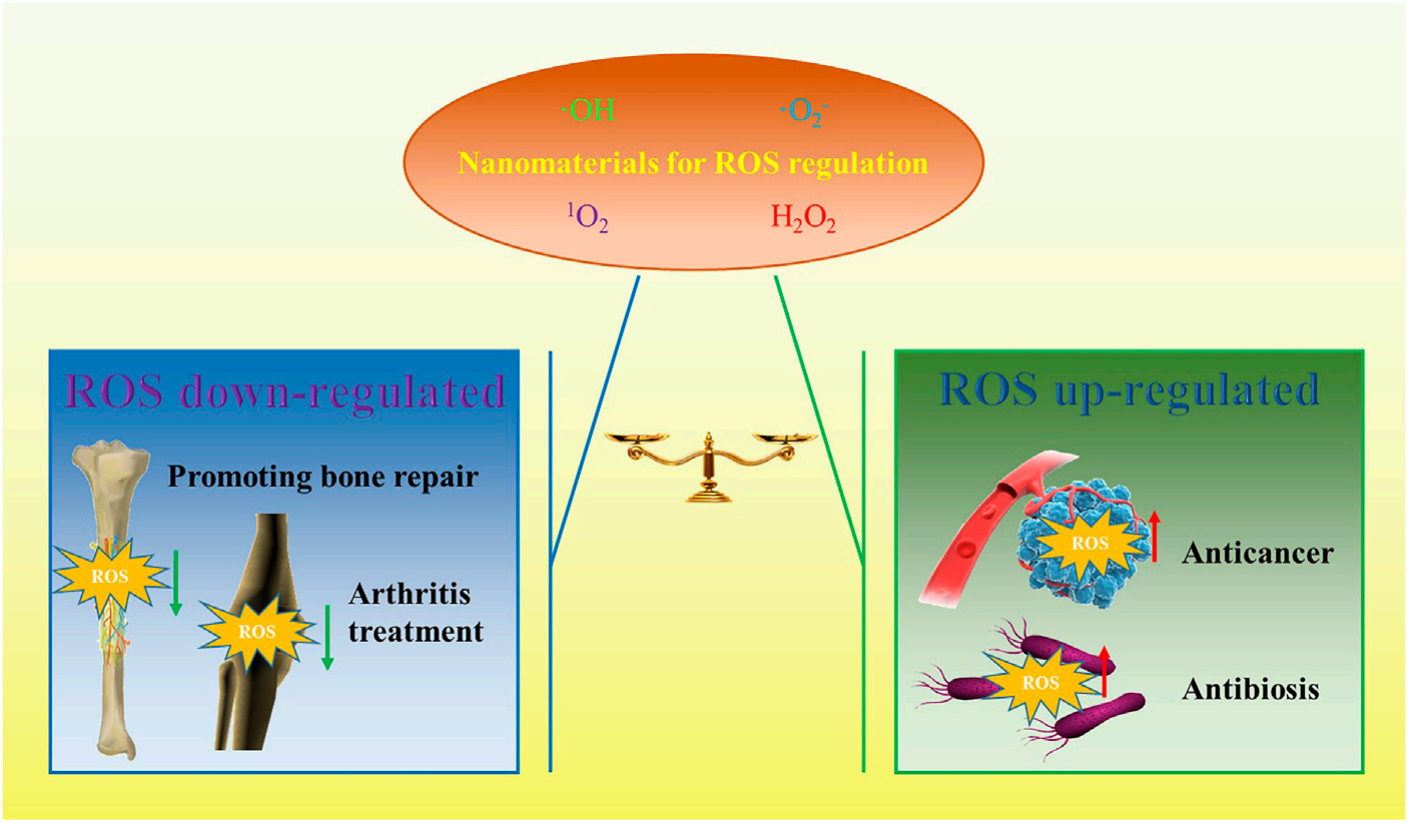
Summary of the application direction of nanomaterials in orthopaedic diseases.

Theoretically, whether ROS acts as a damaging molecule or a signaling molecule depends on the concentration of ROS in cell (He et al., 2021). Therefore, how to accurately regulate ROS is a key issue for ROS as a target or means to treat diseases. In recent years, great progress has been made in the study of ROS regulation. In particular, nanomaterials have been introduced into the field of ROS regulation, which makes the related fields develop rapidly. ROS-based nano therapeutic methods or nano medicines may be applied to regulate the ROS process. This depends on the inherent biophysical and biochemical properties of nanomaterials, such as appropriate size, high surface area, *etc.* Nanomaterials have great potential to modulate ROS compared with traditional drug molecules because they can be modified to achieve lesion targeting, and they can show satisfactory catalytic efficiency due to their special 3D structure. Nanomaterials have made some progress in the treatment of orthopaedic diseases by regulating ROS, such as bone tumors, osteoarthritis, bone defects and so on. Therefore, it is important and necessary to review this topic, which may provide new possibilities for the further development of ROS-based nanomaterials for the treatment of orthopaedic diseases. The search results of existing studies show that photodynamic therapy (PDT), sonodynamic therapy (SDT) and chemodynamic therapy (CDT) are important research directions related to orthopaedic diseases, and the published articles are increasing year by year. Antioxidant therapy or ROS toxicity therapy is the Frontier of nanomaterials in the treatment of orthopaedic diseases, and its application direction in the treatment of orthopaedic diseases is shown in Figure 1.

## 2 An overview of the association of ROS with orthopaedic diseases

ROS that play a key role in the pathogenesis of orthopaedic diseases include H_2_O_2_, hydroxyl radical, superoxide anion, *etc.* Normally, ROS levels are kept low in normal joints (Lepetsos and Papavassiliou, 2016). The occurrence of many orthopaedic diseases may be related to the abnormal increase of ROS level. For patients with osteoarthritis, ROS such as H_2_O_2_, O_2_^−^ are significantly increased in joints (Yao et al., 2019). The over generation of ROS triggers a series of reactions, such as excessive oxidation and DNA damage, which is considered to be one of reasons of chondrocyte loss and tissue damage (Hosseinzadeh et al., 2016). ROS produced by oxidative stress is also closely related to the pathogenesis of osteoporosis. For example, the study of Toker et al. showed that ROS is closely related to diabetic osteoporosis (Toker et al., 2012). An et al. showed that activation of ROS/MAPKs/NF-κB/NLRP3 was the important cause of osteoporosis in Diabetes Mellitus (An et al., 2019). In addition, the occurrence and development of orthopaedic diseases such as rheumatoid arthritis and osteonecrosis of the femoral head are closely related to the abnormal production of ROS((Chen et al., 2022), (Chen et al., 2020)). There are few studies on the relationship between ROS and the occurrence of bone tumors or bone metastases. Whether this association exists still needs to be proved. Some representative studies on ROS and orthopaedic diseases are shown in Table 1.

**TABLE 1:** Some representative studies on the association between ROS and the occurrence and development of orthopaedic diseases.

First author	Publication year	Disease	Mechanism	References
Peng	2021	Glucocorticoids induce femoral head necrosis	Glucocorticoids induce osteoblast apoptosis and autophagy through ROS/JNK/c-Jun signaling pathway	Peng et al. (2021)
An	2019	Diabetic osteoporosis	ROS/MAPKs/NF-κB/NLRP3 pathway activation	An et al. (2019)
Lepetsos	2016	Osteoarthritis	ROS inhibits the generation of cartilage matrix, damages cartilage matrix and leads to chondrocyte apoptosis through multiple signaling pathways	Lepetsos and Papavassiliou, (2016)
Alarcon	2021	Rheumatoid Arthritis	ROS affect T-cell regulation, fibroblast activation, and cause tissue damage	Fresneda Alarcon et al. (2021)
Zheng	2021	Low back pain due to intervertebral disc disease	ROS stimulates nucleus pulposus cells to secrete substance P causing pain	Zheng et al. (2021)

## 3 ROS regulation nanomaterials and the related mechanism of ROS production/elimination

Although the occurrence and development of most orthopaedic diseases are related to the abnormal increase of ROS in intracellular or local microenvironment, both down-regulation and up-regulation of ROS can be used as therapeutic ideas when using nanomaterials to treat different orthopaedic diseases. These has become antioxidant therapy and ROS-mediated cytotoxic therapy, respectively. Like other disease therapy, in the aspect of nanomaterials mediated antioxidant therapy for orthopaedic diseases, ROS scavenging nanomaterials are mainly developed, such as nanomaterials equipped with ROS scavengers or forming structures that catalyze ROS elimination (Huang et al., 2021). The purpose of nanomaterial therapy is to remove excess ROS and maintain normal physiological processes. The vast majority of cells in the body, including osteoblasts and osteoclasts, have their own endogenous ROS scavenging system. However, due to the large amount of ROS produced by various stimuli, the damage will occur when the endogenous ROS scavenging system cannot complete the removal of ROS. At this point, exogenous ROS clearance is very important. At present, many ROS-scavenging nanomaterials have been developed that can effectively alleviate the abnormal ROS increase to stabilize normal physiological functions (Li et al., 2021a). At present, some of them have been used in the research of the treatment of orthopaedic diseases, and some of them are expected to become “new weapons” for the treatment of refractory orthopaedic diseases. Based on the current studies, these nano-ROS scavengers show great potential for the remission and treatment of orthopaedic disease by inhibiting ROS.

Up-regulation of ROS in cells or microenvironment by nanoparticles is called nanoparticle mediated ROS cytotoxic therapy. The main idea of this treatment is to use ROS up-regulated Nano platforms to enhance ROS production at sites such as tumors or bacterial infections, thereby exerting the toxic effects. This treatment idea has been widely recognized in the treatment of osteosarcoma, bacterial infection and so on (Chu et al., 2019; Seo et al., 2021). A large amount of ROS is generated by nanomaterials to form oxidative stress in the cell or microenvironment, which will far exceed the endogenous ROS clearance level of cells or bacteria themselves, thus leading to cell apoptosis or bacterial death. The advantage of using nanoparticles as agents of ROS toxicity is that the nanoparticles can be modified to achieve site-specific and controlled toxicity treatment. At present, there are mainly three kinds of nanoparticles that exert ROS-toxic effects 1): ROS generated by carrying photosensitizer, sonosensitizer and other molecules (Ocsoy et al., 2016; Lee et al., 2021); 2) ROS synthesis catalyzed by a special structure independent of conventional photosensitizer/acoustic sensitizer (Yi et al., 2020); 3) Combined application of the two mechanisms. The common treatment methods of ROS toxicity using nanomaterials include chemodynamic therapy (CDT), photodynamic therapy (PDT), sonodynamic therapy (SDT), *etc.* (McHale et al., 2016; Kwiatkowski et al., 2018; Yu et al., 2021). These treatment ideas have been reflected in the treatment research of related orthopaedic diseases. In this section, we summarize the currently known strategies and approaches for the treatment of ROS-related orthopaedic diseases, including antioxidant therapy and ROS-induced toxicity.

### 3.1 ROS scavenging nanomaterials for antioxidant treatment of orthopaedic diseases

**FIGURE 2:**
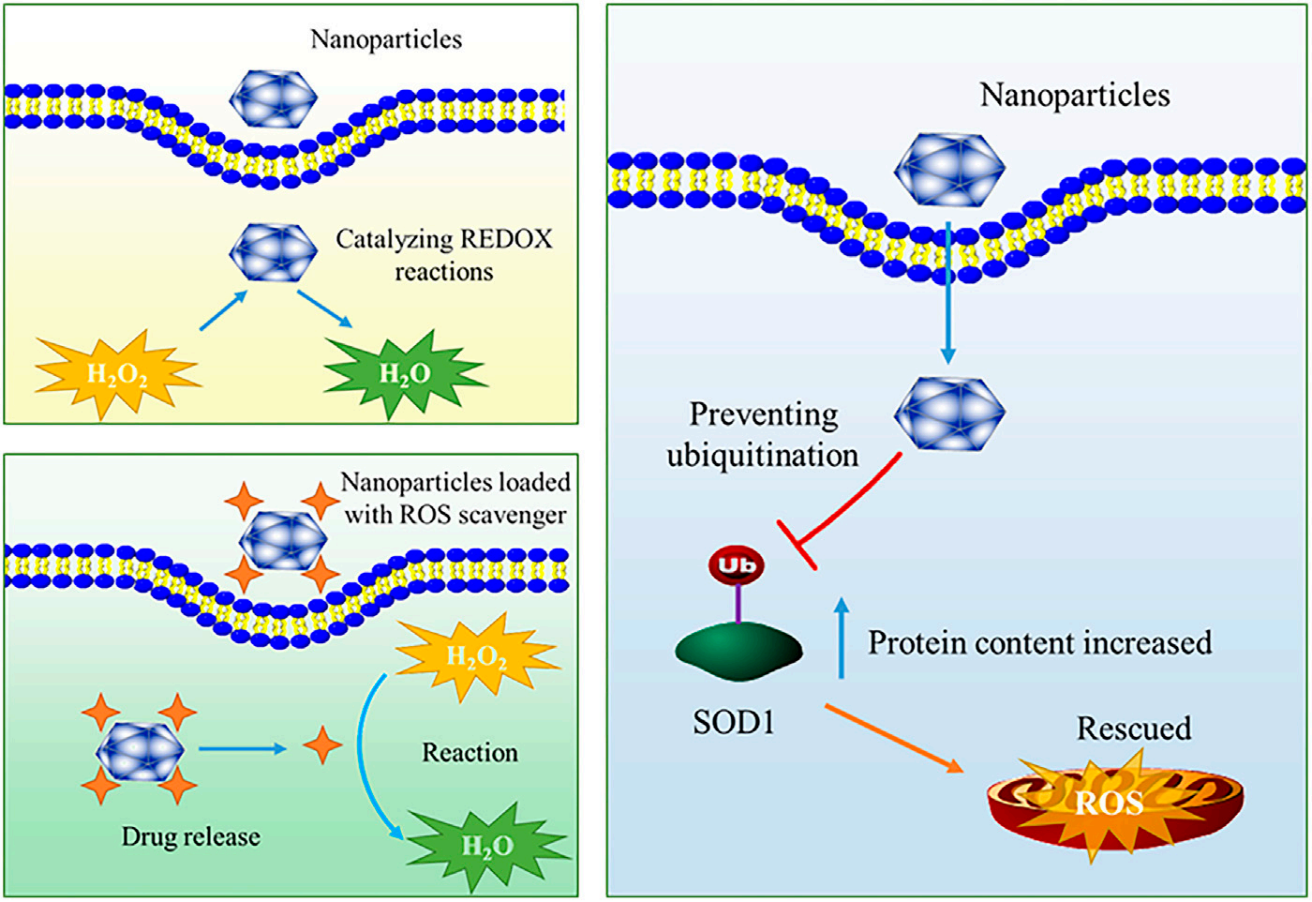
Overview of the mechanism of common ROS scavenging nanoparticles.

As mentioned above, the production of ROS in cells or microenvironment increases significantly under significant stress, and endogenous antioxidants cannot remove all ROS, resulting in serious damage to biomolecules including DNA, lipids and proteins, and triggering a series of reactions such as ER stress and mitochondrial damage related apoptosis (Tian et al., 2020; Tu et al., 2021). This is an important mechanism by which ROS promotes the occurrence and development of orthopaedic diseases. At this time, it is particularly important to introduce exogenous substances to remove ROS. However, it must be admitted that the current drugs used to remove ROS still face toxicity problems, stability problems and difficulties in clinical translation (Zhong et al., 2019). In recent years, with the development of nanotechnology, novel ROS scavenging strategies based on multifunctional nanomaterials have been widely studied in the design of ROS scavengers, providing a new opportunity for the treatment of related orthopaedic diseases. Current studies indicate that a great deal of effort has been invested in the development of ROS-scavenging nanomaterials for the treatment of orthopaedic diseases, some representative studies are shown in Table 2. Nanomaterials can be equipped with ROS scavengers to achieve the purpose of ROS elimination. This approach can improve the lack of targeting and poor water solubility of traditional ROS scavenger drug molecules. In addition to being drug carriers, some nanomaterials have direct elimination effect on ROS, which is usually related to the REDOX reaction ability of nanomaterials themselves or the special catalytic ability of nanomaterials. In addition, some nanomaterials can inhibit ROS production by regulating endogenous ROS scavenging pathway. A schematic illustration of mechanism is summarized in Figure 2. The first two kinds of ROS scavenging nanomaterials are described in detail below.

**TABLE 2:** Representative ROS scavenging nanomaterials for orthopaedic diseases.

First author	Publication year	Representative antioxidant nanomaterials (^a^)	Indications	Related mechanism	References
Kim	2019	MFC-MSNs	Rheumatoid arthritis	MFC-MSNs can synergistically eliminate ROS and produce O_2_, reduce the M1 macrophages, and induce M2 macrophages to participate in RA treatment	Kim et al. (2019)
Yang	2021	FA-AgNPs	Rheumatoid arthritis	FA-AgNPs release Ag^+^ in the presence of GSH, which then scavenge ROS and promote the polarization of M2 macrophages	Yang et al. (2021a)
Lin	2022	HA@M@PB@SIN NPs	Rheumatoid arthritis	Nanoparticles significantly inhibited the abnormal proliferation of fibroblast-like synovial cells by scavenging ROS and inhibiting the proinflammatory cytokines secretion	Lin et al. (2022)
Crivelli	2019	SFNs/CUR	Osteoarthritis	The combined application of curcumin and SFNs showed synergistic antioxidant effect to achieve ROS scavenging	Crivelli et al. (2019)
Kiyani	2019	Zinc oxide nanoparticles	Gouty arthritis	Zinc oxide nanoparticles can play a role in the treatment of gouty arthritis by reducing oxidative stress and ROS level	Kiyani et al. (2019)
Zhong	2019	Dopamine melanin nanoparticles	Osteoarthritis	DM nanoparticles can prevent oxidative stress, reduce ROS and promote autophagy to treat osteoarthritis	Zhong et al. (2019)
Pinna	2021	Ce@MSNs	Osteoporosis	Ce@MSNs could significantly reduce the oxidative stress produced by t-butyl hydroperoxide	Pinna et al. (2021)
Yu	2020	Fe_2_O_3_@PSC nanoparticles	Osteoporosis	Nanoparticles eliminate excessive ROS released after Fe^3+^ stimulation of mitochondria by activating Keap1/Nrf2/HO-1 signaling pathway	Yu et al. (2020)
Zhou	2022	Prussian blue nanoparticles	Intervertebral disc degeneration	PBNPs can stabilize SOD1 protein, improve antioxidant capacity, reduce ROS, and rescue ROS-induced IVDD.	Zhou et al. (2022b)

a The names of nanomaterials are listed here. The specific ingredients or full names are shown in the corresponding references.

#### 3.1.1 Nanomaterials loaded with ROS scavenger

Introduction of ROS scavengers is a common strategy to remove excess ROS in cells. However, these ROS scavengers have inevitable drawbacks, including lack of cell targeting and possible instability. The introduction of nanocarriers may accelerate the further development of ROS scavengers for the treatment of orthopaedic diseases. Nanomaterials as carriers of ROS scavengers can improve the stability of ROS scavengers, and can also give these molecules the ability to target and reduce their side effects (Lee et al., 2019; Khalil et al., 2020). The types of ROS scavengers loaded may include some small molecules of drugs, or some large molecules, such as enzymes that can eliminate ROS.

The use of nanoparticles to deliver small molecules capable of scavenging ROS is a common research idea for the treatment of related orthopaedic diseases. These small molecules are usually involved in REDOX reactions to achieve the purpose of scavenging ROS. However, it is important to point out that this delivery does not necessarily depend on the “loading” process, and ROS scavengers may become part of the nanomaterial. For example, Zhao et al. designed a nanoparticle P (DMA-co-MPC) in the study on the treatment of osteoarthritis, in which DMA is N-(3,4-dihydroxyphenethyl) methacrylamide, which acts as ROS scavenger (Zhao et al., 2020). DMA contains reducing phenolic hydroxyl groups, which may be the reason for its ROS scavenging ability. Through experiments, the authors proved that nanoparticles can regulate inflammation, pain and other related pathways by removing ROS, which may be a potential drug for the treatment of osteoarthritis. Zhou et al. used nanoparticles synthesized by Fe^3+^ and curcumin to treat osteoarthritis through the anti-oxidative stress effect of curcumin (Zhou et al., 2022c). In addition to being loaded with small molecules, nanoparticles can also be loaded with enzymes or short peptides to scavenge ROS to treat orthopaedic diseases. Common enzymes that can remove ROS include SOD and so on (Saxena et al., 2022). Some short peptides, such as GSH, are also involved in the removal of intracellular ROS((Yang et al., 2016)). For example, Gui et al. mentioned a SOD-loaded porous polymer nanoparticle that could be targeted to the synovial membrane as a means of treating osteoarthritis (Gui et al., 2022). Gao et al. also designed a nanomaterial containing SOD as a treatment for arthritis (Gao et al., 2020). To date, the use of nanoparticles to deliver ROS scavengers in the treatment of orthopaedic diseases still has great room for development. We believe that the key is how to design the structure of the nanomaterial carrier so that it could be targeted, low toxicity, and high drug loading ability. The use of engineered liposomes or exosomes for ROS scavenger delivery may be a valuable research direction. As for the ROS scavengers that could be loaded, not only some small molecule ROS scavengers, but also some biological molecules such as coenzyme Q10 may be suitable choices. Further experiments are needed to explore which ROS scavengers have more advantages. It is well known that the use of nanomaterials as the carrier of ROS scavengers indeed makes the application prospect of ROS scavengers more promising, but there is still a certain gap between nanoparticles and clinical practice due to its stability and how to produce them on a large scale.

#### 3.1.2 Nanomaterials with ROS quenching effect independent of traditional ROS scavenging molecules

The aforementioned nanoparticles equipped with ROS scavengers can effectively improve the pharmacokinetics, targeting and other deficiencies of some ROS scavengers. However, they still have some shortcomings, such as stability in circulation. At the same time, the complexity of nanoparticle synthesis also makes the application of nanoparticles equipped with ROS scavenger limited. Therefore, it is necessary to design new antioxidant strategies that do not rely on traditional ROS scavenging molecules. In recent years, more studies have focused on the use of nanomaterials themselves to remove ROS. This kind of nanomaterials often have special structure or inherent catalytic properties, including nanozymes, *etc.* Some nanomaterials that can remove ROS depend on their ability to participate in REDOX reactions, such as dopamine nanomaterials (Newland et al., 2016). Interestingly, some metal oxide nanoparticles seem to exhibit good ROS scavenging ability. For example, cerium oxide nanoparticles are a typical type of nanoparticles with ROS scavenging ability. Vacancy-oxygen defect existing in the cerium oxide nanoparticle shows an advantage in ROS removal (Li et al., 2020c). In their review, Li et al. summarized the synthesis, structure and properties of cerium dioxide nanoparticles in detail, and introduced the mechanism of ROS scavenging by cerium dioxide nanoparticles, as well as some applications in bone tissue engineering (Li et al., 2020c). The ROS scavenging mechanism of nanoparticles fully demonstrates the importance of the microstructure of nanomaterials. Another popular nanoparticle with ROS scavenging ability is MnO_2_ related nanoparticle, which is often studied as a “nanozyme”. Some studies have shown that MnO_2_ nanoparticles have the ability to catalyze the reaction of hydrogen peroxide (Gordijo et al., 2015). In the application study of orthopaedic disease treatment, Chen et al. designed a hollow MnO_2_ nanoparticle with good ROS scavenging ability for the treatment of osteoarthritis (Chen et al., 2021). Kumar et al. mentioned that MnO_2_ nanoparticles can protect cartilage from oxidative stress caused by inflammation (Kumar et al., 2019). Zhu et al. designed a hollow MnO_2_ nanoparticles loaded with TGF-β3 to treat intervertebral disc degeneration, and the authors indicated that the hollow MnO_2_ nanoparticles played an important role in the antioxidation (Zhu et al., 2022). In addition to cerium oxide and manganese dioxide nanoparticles, other metal oxides such as molybdenum oxide and nickel oxide nanoparticles may also be used as functional nanoparticles for ROS scavenging (Mu et al., 2016; Duan et al., 2022). Some of these metal oxide nanoparticles have not been used in orthopaedic disease treatment research, and more relevant exploration can be carried out in the future.

In addition to the above metal oxide nanoparticles, some other types of nanoparticles, such as noble metal nanoparticles (gold, silver, platinum, etc.), polydopamine nanoparticles, etc., may have high activity of ROS scavenging activity (Zhao et al., 2018c; Yang et al., 2018). For example, Sul et al. mentioned in their study that gold nanoparticles can reduce ROS level and affect osteoclast formation (Sul et al., 2010). Nomura et al. showed that Pt nanoparticles could inhibit RANKL-stimulated osteoclast differentiation through ROS scavenging properties (Nomura et al., 2011). It is worth noting that each of these nanoparticles has its own potential applications, for example, the unique microstructure of some metal oxide nanoparticles, the high catalytic efficiency of some metal-organic framework nanomaterials, the specific surface area advantage of noble metal nanoparticles, and the modifiability of some organic nanomaterials. Future research should focus on combining the advantages of different types of nanoparticles to achieve the effect of “learning from each other”. In addition, future studies should focus on the specific mechanism of ROS elimination by nanomaterials, including the possible potential reaction mechanism and influencing factors, so as to provide a solid theoretical basis for ROS elimination of nanomaterials in the treatment of orthopaedic diseases.

### 3.2 Nanomaterials used to generate ROS toxicity for the treatment of orthopaedic diseases

**FIGURE 3:**
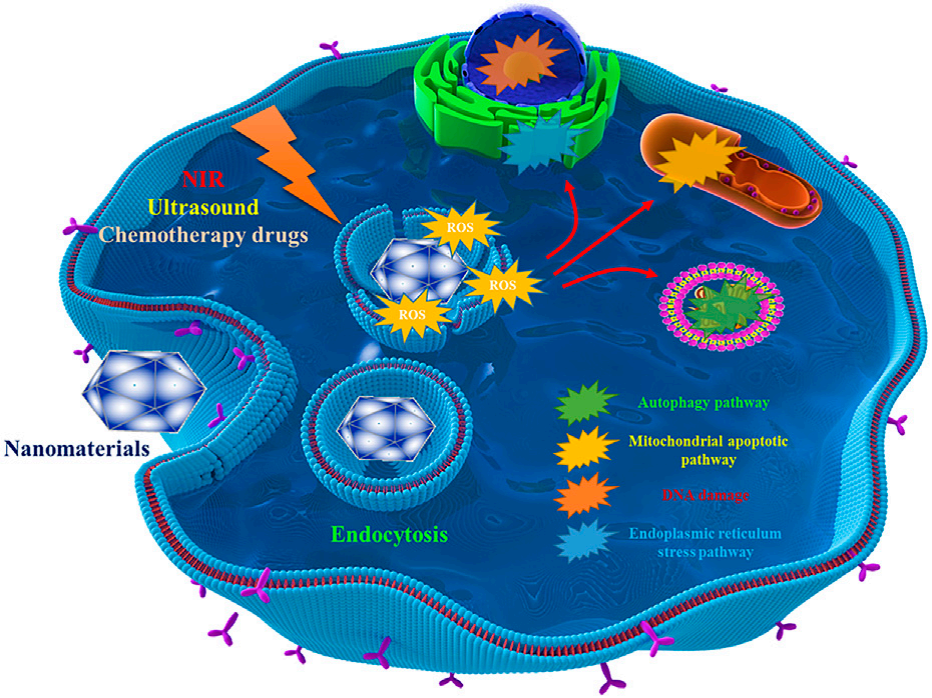
Effects of ROS production-related nanomaterials on intracellular pathways/structures.

ROS is a double-edged sword (Lu et al., 2018). As mentioned above, a certain amount of ROS is usually maintained in cells as a “messenger” for the regulation of some signaling pathways (Checa and Aran, 2020). However, excessive ROS can cause cell death. In the case of normal cells or bacteria, excessive ROS is very difficult to tolerate. This brings a new solution to some abnormal or proliferative orthopaedic diseases, such as bone tumors and infection after orthopaedic surgery. In recent years, the use of nanomaterials to increase the intracellular ROS content to kill target cells has become a popular method. Common ROS mainly include H_2_O_2_, •O_2_^−^, •OH, ^1^O_2_, *etc.*, and most strategies focus on improving these ROS with the help of nanomaterials. Depending on the different stimulating methods, these strategies are often classified as photodynamic therapy, sonodynamic therapy, and chemodynamic therapy (Wang et al., 2021). A large amount of ROS produced can cause DNA damage, endoplasmic reticulum stress and mitochondrial apoptosis, *etc.* ROS can also induce cell apoptosis by influencing other related cell signaling pathways. For example, Huang et al. designed nanomaterials to inhibit autophagy and lead to apoptosis through endoplasmic reticulum stress related to ROS,etc. (Huang et al., 2020). However, it should be pointed out that the regulation of many signaling pathways by ROS has various possibilities, which showing certain complexity, so the regulation of autophagy and other cellular pathways by ROS needs to be careful. The relevant mechanism is shown in Figure 3. Meanwhile, around the topic of ROS generation, the designed nanomaterials need to have some supporting functions, such as increasing the local oxygen content to maintain the continuous supply of ROS((Cheng et al., 2015)). Considering the significance of nanomaterials leading to ROS overload in orthopaedic tumors and orthopaedic surgical infections, in this section we will review the treatment strategies leading to ROS overload by classification according to excitation mode. Some representative ROS-enhanced nanomedicines are listed in Table 3.

**TABLE 3:** Representative nanoparticles stimulated ROS generation and their effects.

First author	Publication year	Representative nanoparticles (^a^)	Indication	Therapeutic modality	Specific related mechanisms	References
Zeng	2021	TPP-PPG@ICG	Drug-resistant osteosarcoma	Synergistic phototherapy	Nanomaterials acts as a photosensitizer to generate ROS after near-infrared laser irradiation	Zeng et al. (2021)
Huang	2020	Drug-carrying PEG-GO-FA/ICG nanoparticle	Osteosarcoma	Combined chemo-photodynamic effects	Through the combination of photodynamic and chemodynamic, the ROS produced may participate in ER stress and induce apoptosis through the JNK/p53/P21 pathway	Huang et al. (2020)
Yuan	2020	AI-MPDA	Biofilm	NO-enhanced photodynamic therapy and low-temperature PTT (≤45°C)	AI-MPDA catalyzes the production of ROS and the release of nitric oxide (NO) through a cascade reaction	Yuan et al. (2020)
Xie	2022	Ti-MOF-based biosafety material	Infection	Synergistic photodynamic and photothermal effects	TiO_2_ nanodots can catalyze the formation of •O_2_^−^ and ^1^O_2_ from H_2_O_2_ and O_2_ under visible light irradiation, respectively	Xie et al. (2022)
Fu	2019	Fe(VI)@HMON-PpIX-LA-PEG	Osteosarcoma	Sonodynamic therapy	Low-energy US triggers the generation of oxygen and ROS, and iron also participates in the generation of ROS.	Fu et al. (2019)
Wu	2021	Au@BTO	Bacterial elimination and wound healing	Sonodynamic therapy	The ROS produced by Au@BTO nanoparticles under ultrasound has high antibacterial efficiency against both Gram-positive and Gram-negative bacteria. At the same time, sonodynamic therapy can also promote wound healing	Wu et al. (2021)
Fu	2022	Rh2@HMnO_2_-AM	Osteosarcoma	Immuno-chemodynamic combined therapeutic	Mn^2+^ in nanomaterials catalyzes hydrogen peroxide to produce hydroxyl radical	Fu et al. (2022)
Yang	2021	Cu_2_O nanoparticles	Bacterial infection	Photothermal/chemodynamic synergistic anti-infective therapy	Cu^+^ in nanomaterials catalyzes hydrogen peroxide to produce hydroxyl radical by Fenton-like reaction. Nanomaterials can also promote wound repair	Yang et al. (2021b)

a The names of nanomaterials are listed here. The specific ingredients or full names are shown in the corresponding references.

#### 3.2.1 Photodynamic therapy

Photodynamic therapy refers to the generation of ROS by photosensitizer under the excitation of a certain wavelength laser to achieve the purpose of disease treatment. Photodynamic therapy was first realized by some drug molecules, including ICG, Ce6 and so on (Ara et al., 2021; Luo et al., 2021). However, similar to ROS scavengers, these photodynamic therapy drug molecules also have a series of problems such as toxicity and lack of targeting (Mitsunaga et al., 2011). The introduction of nanomaterials solves the shortage of existing photodynamic therapy drug molecules and endows photodynamic therapy with more abundant characteristics. In the research of orthopaedic diseases, especially osteosarcoma, it is a common research idea to use nanoparticles to load common photodynamic therapy drug molecules. Zeng et al. designed an ICG-loaded GO nanoparticle and modified it to achieve osteosarcoma aggregation and generate ROS to kill tumor through photodynamic effect (Zeng et al., 2021). Photodynamic therapy can also be combined with chemodynamic therapy to achieve the purpose of combined treatment to kill osteosarcoma. For example, in the study of Huang et al., a graphene equipped with ICG and doxorubicin was designed to target osteosarcoma, and the mechanism of its killing osteosarcoma was studied in detail (Huang et al., 2020). Specifically, ROS produced by photodynamic therapy is the key to enhanced therapy, which can kill osteosarcoma by affecting DNA integrity, autophagy related pathways and apoptosis-related pathways. In addition to the treatment of osteosarcoma, nanoparticles loaded with photodynamic therapeutic drug molecules have also achieved remarkable results in antimicrobial applications. After orthopaedic surgery, the potential infection risk of wound or prosthesis is one of the problems that must be faced by patients and doctors (Backes et al., 2018; Ronin et al., 2022). Among the pathogens that may lead to infection, *Staphylococcus aureus* is the common one, which can form bacterial film. Based on this problem, Yuan et al. designed a nanoparticle equipped with ICG, which could effectively remove biofilms formed by bacteria by photodynamic therapy under NIR irradiation (Yuan et al., 2020). According to the existing research results, nanomaterials equipped with photosensitizer can indeed achieve better photodynamic therapy effect. However, the similar problem faced by nanomaterials equipped with ROS scavengers is the circulation stability of the nanomaterials. Moreover, the loading load of nanomaterials increases after the photosensitizer is installed, which may affect the further modification of nanomaterials or the loading of chemotherapy drugs. Therefore, it is still necessary to design new photosensitive nanomaterials that do not rely on traditional photosensitizers to achieve more efficient photodynamic therapy.

Whether paying attention to the crystal structure or the 2D/3D structure, nanomaterials have the potential to form special catalytic structures. This catalytic potential can be used for ROS generation. There are already some nanomaterials being developed that have photodynamic effects of their own. The design of these novel photodynamic nanomaterials may be based on the structural characteristics of some traditional photosensitizers, such as porphyrins. These structures can also be directly used in integrated nanomaterials. Cai et al. designed a metal-organic framework material Au@MOF that incorporates porphyrin structures to enable photodynamic therapy (Cai et al., 2021). The use of metal-organic framework nanomaterials to catalyze REDOX reactions and generate ROS is one of the research hotspots. Xie et al. designed a biosafe material based on Ti-MOF to achieve antimicrobial resistance (Xie et al., 2022). In the future research of orthopaedic disease treatment, similar nanomaterials can be more modified in the future to achieve more functional integration, such as the integration of anti-tumor and anti-bacterial, *etc.* At the same time, this kind of nanomaterials can be combined with other biological materials such as hydrogels to enrich the function. Based on the summary of existing studies, the study of ROS generation by photodynamic therapy is relatively mature, and its application is increasingly widespread. However, we must also recognize the limitations of photodynamic therapy in orthopaedic diseases. It is important to note that the vast majority of orthopaedic diseases have lesions located deep in the body. However, most of the lasers required in photodynamic therapy, such as 808 nm near-infrared laser, do not show good tissue penetration ability. In addition, because human muscle tissues are rich in heme, showing a darker color, which may indicate that these normal human tissues also have certain photothermal capacity. When stimulated by the 808 nm laser, the temperature of these tissues may increase even though there is no aggregation of nanomaterials, which may cause unexpected tissue damage. This is an important reason why the application of photodynamic research in the treatment of orthopaedic diseases is limited. Therefore, new ways of generating ROS from nanomaterials should be developed.

#### 3.2.2 Sonodynamic therapy

Sonodynamic therapy is a new concept emerging in recent years, which is a good supplement to photodynamic therapy. Sonodynamic therapy is to use ultrasound to stimulate a high concentration of sonosensitizer concentrated in the target cells to produce a large number of ROS to kill the target cells. Here sonosensitizer includes some small molecules, such as IR-780 ((Li et al., 2016)). For deep lesions, sonodynamic therapy is a suitable alternative to photodynamic therapy, because the tissue penetration of ultrasound is better than that of NIR laser, and it is less likely to cause non-specific damage to normal human tissue (Huang et al., 2017; Ouyang et al., 2020). There have been some studies on the application of nanomaterials in the treatment of orthopaedic tumors. For example, Fu et al. used degradable hollow mesoporous silica based nanomaterials as the carrier to obtain a multifunctional nanomaterial with acoustic dynamic effect, which can accumulate in osteosarcoma and generate a large amount of ROS to kill the tumor under the action of ultrasound (Fu et al., 2019). In the aspect of antibacterial treatment, some interesting results have been obtained by utilizing the sonodynamic effect of nanomaterials. For example, Wu et al. developed an Au@BTO nanoparticle based on piezoelectric electronics. By virtue of ultrasound, the electron-hole effect leads to the occurrence of REDOX reactions and the generation of ROS, so as to achieve the purpose of antibacterial and promoting tissue repair (Wu et al., 2021). At present, the research on the use of nanomaterials for the sonodynamic treatment of orthopaedic diseases is not enough, which may be related to the difficulty in the research and development of new materials. The key to the development of sonodynamic therapeutic nanomaterials is the microstructure of the nanomaterials, especially the design of the catalytic core. At present, there is still a lack of sufficient research basis in related fields. In addition, the use of nanomaterials in the sonodynamic treatment of orthopaedic diseases also has potential shortcomings. The first is the targeting and stability of nanomaterials, which is a common problem in the treatment of orthopaedic diseases with biological nanomaterials. Sonodynamic therapeutic nanomaterials require more microstructure, which makes further modification of the nanomaterials may need to be more careful. Furthermore, although ultrasound can reach deep lesions through tissues, attenuation of ultrasound energy must be considered. In addition, in order to ensure the stability of the effect, the selection of additional materials such as ultrasonic coupling agent also needs to be careful.

#### 3.2.3 Chemodynamic therapy

Chemodynamic therapy is a new treatment for cancer in recent years, which is mainly based on Fenton response or Fenton-like response (Li et al., 2021b). Based on the slightly acidic environment of the tumor area, chemodynamic therapeutic nanoparticles can rapidly generate large amounts of ROS by Fenton reaction or Fenton-like reaction, without the need for activation by external energy (such as laser, ultrasound, *etc.*) (Li et al., 2021b). According to the related theory of chemodynamic therapy, nanoparticles that usually activate chemodynamic therapy include necessary metal elements, such as Fe, Cu, *etc.* (Wu et al., 2019; Koo et al., 2022). Chemodynamic therapy is often used in conjunction with other therapies such as photothermal, photodynamic, or immunotherapy to achieve synergistic effects. In the treatment of orthopaedic related diseases, chemodynamic therapy has more advantages for malignant bone tumors. Fu et al. designed a nanomaterial relying on manganese ions to catalyze the generation of hydroxyl radicals from hydrogen peroxide (Fu et al., 2022). They combined immunotherapy with chemodynamic therapy and provided a new idea for the treatment of osteosarcoma. On the antimicrobial side, chemodynamic therapy also shows more promise when used in conjunction with other therapies. Yang et al. developed a Cu_2_O based nanoparticle that catalyzes Fenton-like reactions to generate hydroxyl radicals with strong antimicrobial activity, while also promoting tissue repair (Yang et al., 2021b). The development of such nanoparticles is of great significance for the treatment of methicillin-resistant *Staphylococcus aureus*, a common intractable pathogen in wound infection, and for the treatment innovation of orthopaedic trauma. Combined with the theoretical basis of Chemodynamic therapy, when the microenvironment of the lesion is acidic, chemodynamic therapy will show great advantages. When the focal environment is weakly alkaline (usually normal tissue), the rate of Fenton/Fenton-like response is limited, which may be indicative of limited damage to normal tissue by chemodynamic therapy. However, nanomaterials undergoing chemodynamic therapy are mostly used as catalysts, which can continuously produce ROS. When combined with chemotherapy drugs or other therapies such as photothermal and photodynamic therapies, they often achieve complementary and synergistic effects by affecting autophagy, mitochondrial apoptosis, DNA damage and other pathways. However, chemodynamic therapy also needs to be concerned, that is, for osteosarcoma, the lesion may present a state of hypoxia locally, which is also a common feature of some solid tumors (Zhang et al., 2020). Hypoxic environments may affect ROS production. Therefore, the future design of related nanomaterials should also be taken into account the problem of oxygen supply.

## 4 Considerations for the design of nanomaterials related to REDOX reactions

In conclusion, the related nanomaterials based on ROS-regulated REDOX reactions show great application value for the treatment of orthopaedic diseases. We reviewed the relevant studies and believe that the following considerations should be paid attention to in the future research and development of nanomaterials for orthopaedic diagnosis and treatment:

1) Design nanomaterials with a clear purpose. Nanomaterials based on ROS regulation should focus on ROS generation or elimination. In the process of designing nanomaterials, the structure of nanomaterials and the corresponding function should be considered, rather than blindly designing a material to verify the function. In general, the catalytic core or functional unit of the nanomaterial should be given sufficient attention.
2) Design nanomaterials based on clinical and pathological features. Different orthopaedic diseases have different etiology, location, microenvironment characteristics of local lesions, and special gene expression/signal pathway changes. These characteristics should be fully integrated in the design of nanomaterials.
3) Good biocompatibility and circulation stability. This is a common standard for biological nanomaterials, which is the first step of nanomaterials research and development to application. Since the vast majority of nanomaterials used in orthopaedic diseases require systemic administration, any relevant research needs to verify the toxicity and circulatory stability of the developed nanomaterials. Studies of toxicity and circulatory stability *in vivo* are essential. If there is a potential toxicity, the study should be discontinued immediately. If possible, nanomaterials of the corresponding structure and composition should be reported by means of reports.
4) “Simple” design. The design of biological nanomaterials should aim to perform the relevant function in the simplest possible structure. The complex multilayer structure, repeated drug loading and modification of various macromolecules may cause the structure of nanomaterials to be confused and unstable, and the functional study of nanomaterials will become more complicated.
5) Pay attention to the combination of multiple treatment methods. The combination of treatment methods is the common goal of orthopaedic disease treatment. Nanomaterials can be loaded with therapeutic drugs and control their release in cells, and enhance the efficacy of therapeutic drugs by regulating ROS. At the same time, the combination of ROS-based nanomaterials and other treatment methods, such as radiotherapy and photothermal therapy, can also achieve better results. Under the premise of meeting the design principle of “simplification”, the combination of multiple treatment methods may achieve better treatment effect.

## 5 Reflection and prospect

It is undeniable that the occurrence or development of the vast majority of orthopaedic diseases are related to the disorder of local metabolic microenvironment. Taking some key metabolic reaction products such as ROS as disease regulatory targets has become an important idea for the prevention and treatment of orthopaedic diseases. In recent years, nanomaterials related to ROS regulation have been developed more. Because the preparation process is mature and can be reasonably modified, nanomaterials are considered as the symbol of precision, efficiency and safety in the treatment of orthopaedic diseases. We believe that the synthesis of nanomaterials with therapeutic function is the most promising solution for some refractory orthopaedic related diseases, such as malignant primary bone tumors, bone metastases, and wound infection after orthopaedic surgery. Although many nanomaterials still face many problems, such as lack of specific targets and so on. There are some problems in photodynamic therapy and acoustic dynamic therapy, such as insufficient penetration and energy attenuation. But these problems have a chance to be solved. The first is to strengthen the mechanism research of orthopaedic related diseases, find the specific pathways of diseases through high-throughput sequencing and other means, and explore the specific targets of diseases. At the same time, research in the field of materials science should be strengthened, especially the development of suitable acoustic/laser dielectric materials for therapeutic use.

However, there are still some problems worth reflecting upon when reviewing the existing related researches. For example, how to reasonably judge the specific regulatory effect of a structure or component on ROS and its influencing factors is a key issue. Interestingly, we often see studies in which different research come to different conclusions about the same structure or composition. For example, for the regulation of ROS by curcumin, some studies propose that it is a ROS scavenger, while others show that it is a molecule that promotes ROS production (Zeng et al., 2020; Wu et al., 2022). What are the influencing factors in this need to be clarified, which may include concentration, action time and so on. In addition, the non-specific phagocytosis and targeting effects of nanomaterials still need to be discussed. We have to admit that the application of biological nanomaterials in the treatment of orthopaedic diseases is still relatively not wide, one of the important reasons is that nanomaterials in the circulation may still be non-specific phagocytosed by the liver and spleen, so the targeting effect is limited. While reviewing published studies, we found that some of the proposed nanoparticles seem to have shown better targeting and very low nonspecific phagocytosis, and the reasons for this should be further explored and elucidated. Finally, ethics is an issue that must be considered. Some structures of nanomaterials mentioned in some studies may have certain risks, including nanomaterials synthesized by membrane of bacteria and viruses, *etc.* The safety of nanomaterials should be fully evaluated during the study, including whether they may activate unexpected immune reactions, *etc.* When similar nanomaterials are prepared for clinical application in the future, the ethical review and pre-clinical trials must be more rigorous. However, more reflection is for the better development. We always believe that nanomaterials will play an increasingly important role in the treatment of orthopaedic diseases in the future.
